# Fully Physically Crosslinked Conductive Hydrogel with Ultrastretchability, Transparency, and Self-Healing Properties for Strain Sensors

**DOI:** 10.3390/ma16196491

**Published:** 2023-09-29

**Authors:** Feng Ji, Pengbo Shang, Yingkai Lai, Jinmei Wang, Guangcai Zhang, Dengchao Lin, Jing Xu, Daniu Cai, Zhihui Qin

**Affiliations:** 1 College of Chemical Engineering and Materials Science, Quanzhou Normal University, Quanzhou 362000, China; 2 College of Materials Science and Engineering, Fuzhou University, Fuzhou 350116, China; 3 Shenzhen Institute for Drug Control, Shenzhen 518057, China; 4 State Key Laboratory of Metastable Materials Science and Technology, School of Environmental and Chemical Engineering, Yanshan University, Qinhuangdao 066004, China

**Keywords:** conductive hydrogel, carrageenan, self-healing, strain sensor

## Abstract

Currently, conductive hydrogels have received great attention as flexible strain sensors. However, the preparation of such sensors with integrated stretchability, transparency, and self-healing properties into one gel through a simple method still remains a huge challenge. Here, a fully physically crosslinked double network hydrogel was developed based on poly(hydroxyethyl acrylamide) (PHEAA) and κ-carrageenan (Car). The driving forces for physical gelation were hydrogen bonds, ion bonding, and electrostatic interactions. The resultant PHEAA-Car hydrogel displayed stretchability (1145%) and optical transparency (92%). Meanwhile, the PHEAA-Car hydrogel exhibited a self-healing property at 25 °C. Additionally, the PHEAA-Car hydrogel-based strain sensor could monitor different joint movements. Based on the above functions, the PHEAA-Car hydrogel can be applied in flexible strain sensors.

## 1. Introduction

Recently, flexible skin-like strain sensors have gained great attention in the field of human motion detection, health monitoring, and intelligent robotics [1,2,3,4]. These applications require strain sensors to be stretchable, sensitive, and easily self-healing after damage [5]. Lots of materials can be used to fabricate elastomers or hydrogels for strain sensors. Usually, an elastomer-based strain sensor is constructed by integrating conductive nanofillers (e.g., graphene, carbon black, metallic nanomaterials, etc.) into a special polymer matrix (e.g., rubbers (natural rubber and thermoplastic elastomers), poly(dimethylsiloxane), ecoflex, etc.) [6,7,8,9,10]. For example, Zhan et al. fabricated a multi-sensing elastomer based on a reduced graphene oxide/natural rubber, and the resulting wearable sensors could monitor human motions in real time [11]. Similarly, Qu et al. constructed a sensing elastomer based on polydopamine, nitrile rubber, and carbon black, which could detect different joint motions [12]. Unfortunately, because these conductive nanofillers are non-transparent, the transparency of these kinds of sensors is always unsatisfactory, which will restrict their use in visualization [13,14]. Furthermore, these elastomer-based sensors often show poor stretchability and are much harder than the soft human skin, which will limit their application in human motion detection.

Compared with elastomer-based sensors, conductive hydrogel-based sensors seem to be more popular among researchers due to their flexibility, conductivity, and controlled transparency. Hydrogels with conductivity can convert mechanical deformation into electrical signals. Hydrogels with transparency can allow the devices to be visualized in practical applications. In general, there are two conventional strategies to endow hydrogels with conductivity. The first strategy is to introduce conductive nanomaterials or polymers (e.g., graphene, carbon nanotubes, carbon fibers, polyaniline, poly (3,4-ethylenedioxythiophene): polystyrene sulfonate, polypyrrole, polythiophene, etc.) into hydrogels [15,16,17]. However, since these conductive materials themselves are non-transparent, the transparency of these kinds of hydrogels is as unsatisfactory as elastomer-based sensors. Compared to the above conductive hydrogels, ionic conductive hydrogels, by introducing inorganic salt ions (e.g., LiCl, KCl, NaCl, Fe^3+^, Ca^2+^, etc.) into the hydrogels, are more popular, owing to their intrinsic transparency [18,19,20]. For instance, Zhu et al. constructed an ionized polyacrylamide (PAAm)-carboxymethyl chitosan (CMCS)-LiCl hydrogel-based strain sensor with transparency [21]. Similarly, Yang et al. prepared an ionized PAAm-agarose (Agar)-LiCl hydrogel-based strain sensor with transparency by combining a chemically linked PAAm network and a hydrogen bond-associated Agar network [22]. Unfortunately, these hydrogel-based strain sensors could not heal at room temperature once damaged, owing to the irreversible breakages of the chemically linked network, which would decrease their service life.

Up to now, researchers have conducted many explorations to solve these issues, and it has been found that constructing fully physically linked hydrogels may be a good way to endow hydrogels with self-healing performances, since physical interactions are reversible [23,24,25,26]. For example, based on hydrogen bonds and hydrophobic interactions, Liu et al. prepared a fully physically linked hydrogel with a remarkable self-healing performance by incorporating polystyrene-co-poly(N,N-dimethylacrylamide) microspheres into hydrophobic association gel networks [27].

Hydroxyethylacrylamide (HEAA) is a monomer containing two hydrogen-bond donors in the form of free hydroxyl and amide groups. It can be easily polymerized to poly(N-hydroxyethylacrylamide) (PHEAA) in a water solution. Unlike PAAm, hydrogen bonds among PHEAA molecules can form a PHEAA network even in the absence of a chemical crosslinker. Meanwhile, as the physical interactions in the PHEAA network are reversible, a PHEAA-based hydrogel often possesses a self-healing ability by selecting the proper gel matrix. Thus, HEAA has received increasing attention in the preparation of self-healing hydrogels. For example, Zhang et al. synthesized a physically linked poly(hydroxyethyl acrylamide-co-(3-sulfopropyl)-N-methacryloylamidopropyl-N,N-dimethylammonium betaine))-poly(3,4-ethylenedioxythiophene):poly(styrenesulfonate) hydrogel with an outstanding self-healing ability based on hydrogen bonds and electrostatic interactions [28]. However, the above hydrogel was non-transparent since the conductive polymer was non-transparent. Similarly, based on hydrogen bond interactions, our group synthesized a physically linked poly(hydroxyethyl acrylamide-gelatin-glycerin-lithium chloride hydrogel with a self-healing ability [29]. Nevertheless, the above hydrogel was non-transparent since the gelatin network was non-transparent. Although several advances have been made in hydrogel sensors, constructing PHEAA-based hydrogels with transparency and self-healing properties is still challenging work.

κ-carrageenan (Car) is a linear sulfated polysaccharide extracted from red seaweeds, which has good biodegradability and non-toxicity [30]. κ-carrageenan can form thermo-reversible hydrogels by a heating–cooling process. At a high temperature, the broken Car hydrogel can turn to a sol state, and after cooling, it can heal and reform to a bulk hydrogel. The thermo-reversible property of κ-carrageenan can endow Car hydrogels with a self-healing ability at high temperatures [31,32]. For instance, Liu et al. prepared a double network PAAm-Car hydrogel by combining a physically cross-linked Car network and a covalently cross-linked PAAm network, and the maximum tensile strain of the healed PAAm-Car hydrogel was lower than 83% at 90 °C for 2 h [33]. Similarly, Wu et al. also prepared a double network PAAm-Car hydrogel by combining a physically cross-linked Car network and a covalently cross-linked PAAm network, and the maximum tensile strain of the healed PAAm-Car hydrogel was 52% even at 95 °C for 30 min [34]. However, hydrogels inevitably lose water at high temperatures, which might affect their mechanical and electrical properties. Therefore, hydrogels that can heal at room temperature are more desirable. Furthermore, the introduction of Car in the gel can improve the mechanical properties due to the rigid nature of Car [33,34].

Herein, in this work, we selected PHEAA and Car as the gel matrix to construct a fully physically crosslinked PHEAA-Car hydrogel. It is predicted that the PHEAA-Car hydrogel will have stretchability, transparency, and self-healing performances at 25 °C. The driving forces for physical gelation were hydrogen bonds, ion bonding, and electrostatic interactions. Meanwhile, the incorporation of K^+^ and Cl^−^ ions gave the PHEAA-Car hydrogel ionic conductivity. More importantly, the prepared PHEAA-Car hydrogel could detect different joint movements. This research provides a new idea for multifunctional flexible strain sensors as the next generation of wearable devices.

## 2. Materials and Methods

### 2.1. Materials

The Hydroxyethyl acrylamide (HEAA), potassium chloride (KCl), and 2-hydroxy-2-methyl-1-[4-(2-hydroxyethoxy) phenyl] 1-acetone (UV-initiator, Irgacure 2959) were purchased from Aladdin (Shanghai, China). The κ-carrageenan (Car) was obtained from Sigma-Aldrich (Shanghai, China). All other reagents were used as received.

### 2.2. Fabrication of Hydrogels

The PHEAA-Car hydrogels were prepared according to the following method. First, all reactants containing HEAA, Irgacure 2959 (1 mol% relative to HEAA), KCl, Car, and water were added into a three-neck bottle and stirred mechanically at 95 °C for 1 h. Then, the mixed solution was injected into a mold with a pre-heated syringe and then cooled at 4 °C for 1 h. Afterward, the mold was placed under a UV lamp (8 W) for 2 h to carry out the photopolymerization reaction of HEAA. Finally, PHEAA-Car hydrogels were obtained by removing them from the mold. To obtain PHEAA-Car hydrogels with different compositions, the weight ratios of PHEAA to Car and KCl to Car were adjusted. The composition of PHEAA-Car hydrogels is referred to as H_x_C_y_K_z_, where H, C, and K represent the HEAA, Car, and KCl, respectively. x and y represent the weight ratio (wt%) of HEAA and Car in the pre-polymerization solution, respectively. z is the weight ratio of KCl to Car. For example, H_37_C_3_K_6_ means the concentration of HEAA was 37 wt%, the concentration of Car was 3 wt%, and the weight ratio of KCl to Car was 6% in feed.

### 2.3. Characterizations

The attenuated total reflectance Fourier transform infrared (ATR-FTIR) spectra was recorded (2500–700 cm^−1^) on an FTIR spectrometer (Thermo Nicolet iS10, Waltham, MA, USA) after lyophilizing the gel samples.

The transmittance tests of the PHEAA-Car hydrogel in the range of 400 to 800 nm were performed using a UV-Vis spectrophotometer (PerkinElmer, Waltham, MA, USA). The hydrogel samples for the test were 2 mm thick. The test background was air.

The morphology of the hydrogels was observed by a scanning electron microscope. Before the test, the gel samples were frozen by liquid nitrogen and then lyophilized by a vacuum freeze dryer.

### 2.4. Mechanical Test of Hydrogels

Tensile tests were carried out on the dumbbell-shaped hydrogel samples (30 mm × 2 mm × 2 mm) using a tensile machine (Instron 3369, Norwood, MA, USA). The hydrogel was stretched at the velocity of 100 mm/min. Referring to our previous literature report, the elastic modulus, tensile stress, and the elongation were calculated [26]. For test accuracy, each set of test data is an average of seven trials.

### 2.5. Self-Healing Test of Hydrogels

The PHEAA-Car hydrogels with a rectangular shape were cut into two equal parts with a surgical blade from the middle. Next, the fracture surfaces were put together and then stored at 25 °C or 95 °C for a defined time. Finally, the tensile tests of the healed PHEAA-Car hydrogel were carried out. The healing efficiency (HE) was evaluated by HE = σ_t_/(σ_0_ × 100%), where σ_0_ and σ_t_ correspond to the tensile stress of the initial and after-healing PHEAA-Car hydrogel, respectively.

In order to take photos, some of the hydrogel samples were dyed with rhodamine B (1 wt%) and methylene blue (1 wt%) for a few seconds, and then the cut surfaces were brought together to form contact at 25 °C for 12 h. Subsequently, the hydrogel samples were taken out for self-supporting and a holding test.

### 2.6. Sensing Performance Test and Application of Hydrogel-Based Strain Sensor

The PHEAA-Car hydrogel-based strain sensor was assembled with conductive PHEAA-Car hydrogels with a size of 5.0 × 1.0 × 2 cm^3^ and two copper tapes. The copper tapes were used as electrodes at both sides of the PHEAA-Car hydrogel to connect the hydrogel with output copper wires. The voltage was fixed to 3 V in all the electrical tests.

The conductivity (σ, S cm^−1^) values of the PHEAA-Car hydrogel were evaluated by the linear sweep voltammetry method, according to the formula *σ* = *L*/(*R* × *S*), through an electrochemical workstation (Vertex C, IVIUM Tech, Eindhoven, The Netherlands), where *L* means the distance between adjacent electrodes, *R* represents the resistance of the gel sample, and *S* is the cross-sectional area of the gel sample.

For the cyclic tensile loading–unloading measurements, the two ends of the hydrogel were separately connected to the upper and lower fixture of the tensile machine, and meanwhile, the real-time electrical signal was recorded by the same electrochemical workstation when the hydrogel underwent deformation.

The gauge factor (GF) is defined as $GF=\left(\Delta R/R_{0}\right)/\varepsilon$, where ∆*R*⁄*R*_0_ are the relative resistance changes and ε is the strain generated by the hydrogel.

For monitoring human movements, the hydrogel-based strain sensor was fixed onto the specific parts of the volunteer. At the same time, the electrochemical workstation was used to detect the real-time electrical signals.

## 3. Results

### 3.1. Preparation of Hydrogel

**Figure 1:**
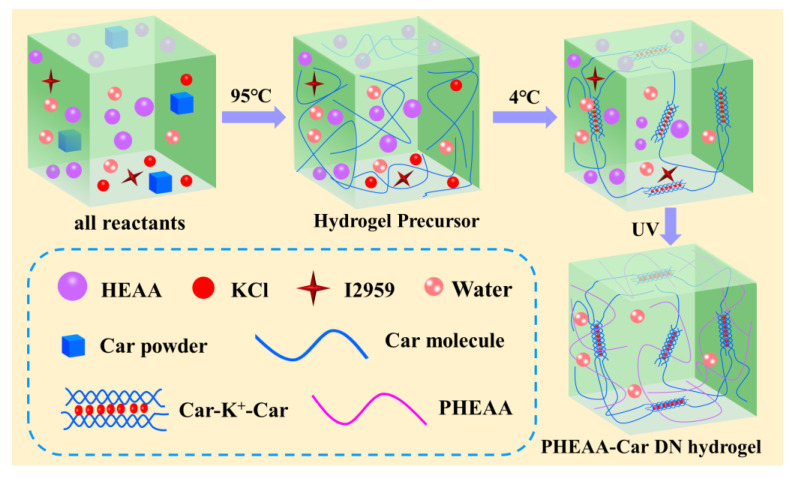
Preparation procedure of PHEAA-Car hydrogel.

Figure 1 illustrates the fabrication process of the PHEAA-Car hydrogel. At first, the HEAA, Irgacure 2959, Car, KCl, and water were stirred mechanically at 95 °C for 1 h to form a homogeneous solution. Next, the solution was cooled to 4 °C for 1 h. At this point, a Car network was formed by the ion bonding and electrostatic interaction of K^+^ ions with κ-carrageenan combined with a hydrogen bond-associated double helix. After the first physically crosslinked Car network was formed, ultraviolet light was applied to initiate the polymerization of HEAA to produce the second PHEAA network. Finally, the PHEAA-Car hydrogel was obtained.

### 3.2. Structure and Morphology Characterization of Hydrogel

**Figure 2:**
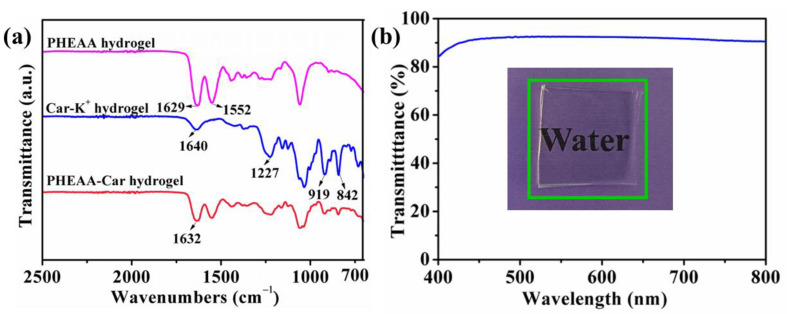
The (**a**) ATR-FTIR and (**b**) transparency of PHEAA-Car hydrogel.

ATR-FTIR spectra were carried out to research the interactions in the hydrogel (Figure 2a). In the spectrum of the PHEAA hydrogel, the peaks of 1629 cm^−1^ and 1552 cm^−1^ were assigned to the C=O stretching vibration and N-H bending vibration, respectively [35]. In the spectrum of the Car-K^+^ hydrogel, the peak at 1640 cm^−1^ corresponded to the C=O stretching. The peak at 1227 cm^−1^ was assigned to the S=O vibration of the sulfate ester (O=S=O) [36]. In addition, the peaks at 919 cm^−1^ and 842 cm^−1^ were attributed to the stretch vibrations of 3,6-dehydrated galactose (C-O-C) and galactose-4-sulfate (C-O-S), respectively [37]. Compared with the PHEAA and Car-K^+^ hydrogels, no new peaks appeared in the spectrum of the PHEAA-Car hydrogel, indicating that no chemical reactions occurred. Meanwhile, a comparison of the spectra of the PHEAA hydrogel and PHEAA-Car hydrogel shows that the stretching vibration of the C=O in the PHEAA hydrogel was shifted to 1632 cm^−1^ from 1629 cm^−1^ in the PHEAA-Car hydrogel, confirming that hydrogen bonds were formed between the Car-K^+^ and PHEAA molecules. Moreover, the PHEAA-Car hydrogel possessed good transparency. As displayed in Figure 2b, the word “Water” under the PHEAA-Car hydrogel can be clearly observed. The transmittance value of the PHEAA-Car hydrogel was around 92% at a 500 nm wavelength.

**Figure 3:**
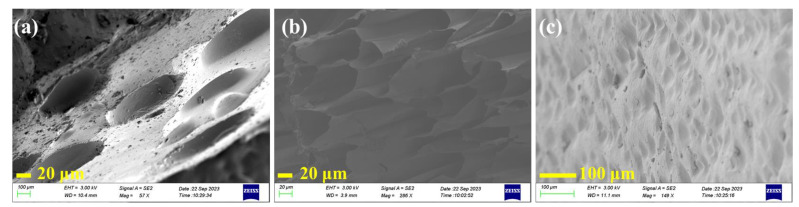
SEM images of (**a**) PHEAA (40 wt%) hydrogel, (**b**) Car (3 wt%) hydrogel, and (**c**) H_37_C_3_K_6_ hydrogel.

In parallel, the morphologies of the hydrogels were investigated by SEM. As shown in Figure 3, the PHEAA hydrogel showed a loose pore structure at a cross-section with a pore size of ca. 85 μm at the long axis, while the Car hydrogel exhibited a uniform pore structure at a cross-section with pore size of ca. 60 μm. Unlike the PHEAA and Car hydrogels, the H_37_C_3_K_6_ hydrogel appeared with a dense porous structure, with pore size of ca. 20 μm, which indicated that there existed interactions between and within the two networks.

### 3.3. Mechanical Property of Hydrogels

**Figure 4:**
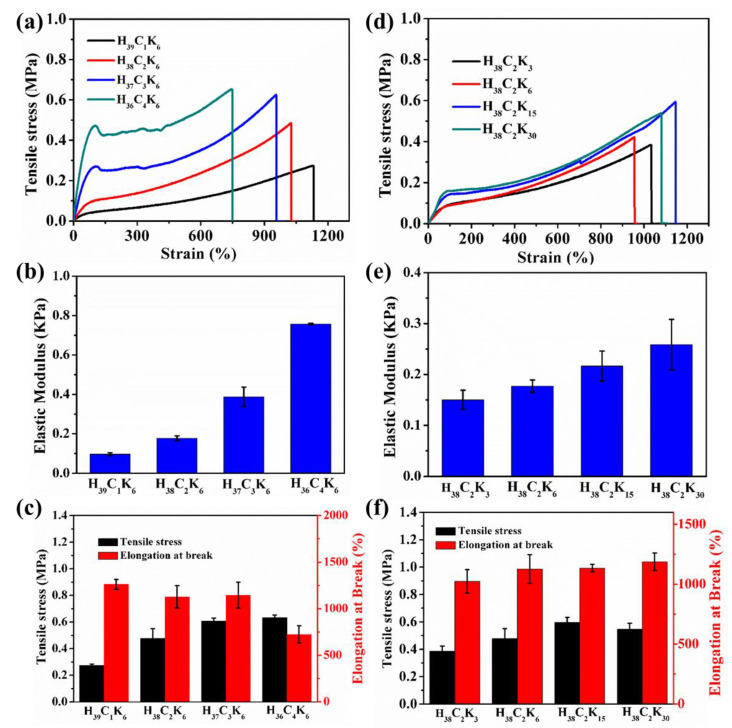
The (**a**) tensile curves, (**b**) elastic modulus, (**c**) tensile stress, and elongation at break of PHEAA-Car hydrogel with different weight ratios of PHEAA to Car. The (**d**) tensile curves, (**e**) elastic modulus, (**f**) tensile stress, and elongation at break of PHEAA-Car hydrogel with different weight ratios of KCl to Car.

The gel composition (e.g., the weight ratio of PHEAA to Car and KCl concentration) had an effect on the mechanical performance of the PHEAA-Car hydrogel. The weight ratio of PHEAA to Car was an important factor to control the mechanical properties. The elastic modulus and tensile stress rapidly increased by increasing the weight ratios of PHEAA to Car from 39:1 to 9:1 (Figure 4a–c). When the weight ratio of PHEAA to Car was 39:1, the elastic modulus and tensile stress of the PHEAA-Car hydrogel were 0.10 MPa and 0.37 MPa, respectively. When the weight ratio of PHEAA to Car increased to 9:1, the elastic modulus and tensile stress of the PHEAA-Car hydrogel rapidly increased to 0.75 MPa and 0.63 MPa, respectively. The higher Car content improved the mechanical properties due to the rigid nature of Car [38]. Moreover, by increasing the weight ratio of PHEAA to Car from 39:1 to 9:1, the elongation at break of the PHEAA-Car hydrogel decreased from 1261% to 724%, suggesting that the increase of Car content decreased the flexibility of the PHEAA-Car hydrogel.

**Figure 5:**
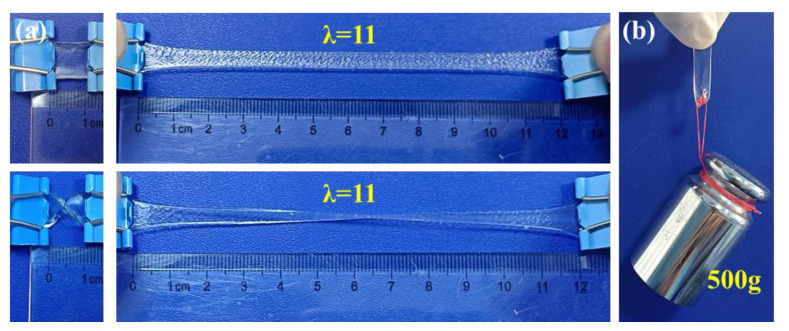
Photographs of H_37_C_3_K_6_ hydrogel showing (**a**) stretching and curly stretching and (**b**) holding a 500 g weight.

Besides the weight ratio of PHEAA to Car, the weight ratio of KCl to Car also influenced the mechanical property of the PHEAA-Car hydrogel (Figure 4d–f). When the weight ratio of KCl to Car increased from 3 wt% to 30 wt%, the elastic modulus of the PHEAA-Car hydrogel monotonically increased from 0.15 MPa to 0.26 MPa. This result suggests that the increase of the KCl content induced high crosslink density. However, by increasing the weight ratio of KCl to Car from 3 wt% to 30 wt%, the tensile stress first increased and then decreased. The maximum tensile stress (0.60 MPa) was achieved at the weight ratio of 15 wt%. This result indicates that the over-crosslinking of K^+^ reduced the number of hydrogen bonds between PHEAA and Car [39]. Meanwhile, all the PHEAA-Car hydrogels possessed high elongation at break in the range of 1023–1187%. Figure 5 vividly shows that the PHEAA-Car hydrogel was flexible, and it could be stretched to 11 times its original length. Moreover, the PHEAA-Car hydrogel could hold 500 g in weight.

**Figure 6:**
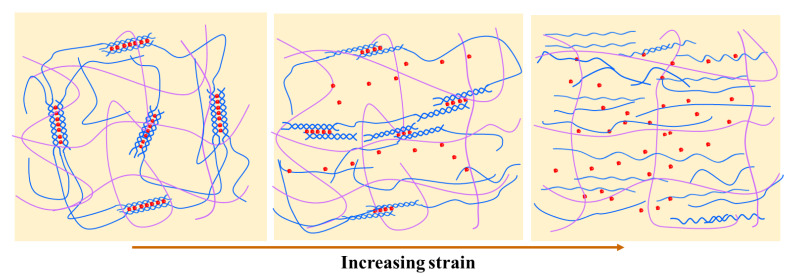
Schematics for the tensile fracture process of PHEAA- Car hydrogel.

Many natural polymers (e.g., agarose, carrageenan, gelatin, etc.) were used as one network to construct the double network hydrogel, because the physical bonds (e.g., ionic bonds, hydrogen bonds, etc.) of the above network are generally weaker than the other network of the hydrogel. Thus, these physical bonds could build an energy dissipation mechanism by breaking themselves instead of breaking the main framework of the hydrogel, resulting in great strength for the whole double network hydrogel. For the PHEAA-Car double network hydrogel, the ionically crosslinked double-helical aggregates and the hydrogen bond cross-linked double helices in the Car network were broken to serve as sacrificial bonds during extension. The schematics for the tensile fracture process of the PHEAA-Car hydrogel are displayed in Figure 6.

### 3.4. Self-Healing Property of Hydrogel

**Figure 7:**
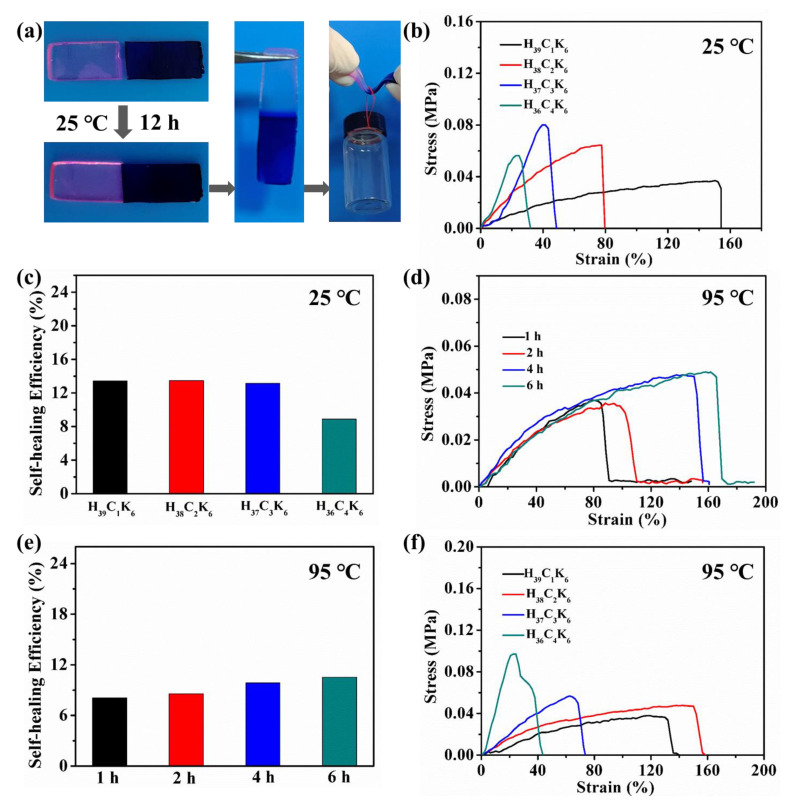
(**a**) The self-healed H_38_C_2_K_6_ hydrogel could hold a weight of 18 g. (**b**) Tensile curve and (**c**) self-healing efficiency of healed PHEAA-Car hydrogel with different weight ratios of PHEAA to Car at 25 °C for 12 h. (**d**) Tensile curve and (**e**) self-healing efficiency of healed H_38_C_2_K_6_ hydrogel with different healing times at 95 °C. (**f**) Tensile curve of PHEAA-Car hydrogel with different weight ratios of PHEAA to Car at 95 °C for 4 h.

Owing to the reversible non-covalent interactions, the PHEAA-Car hydrogel may possess a self-healing property. As shown in Figure 7a, when two parts of the PHEAA-Car hydrogel (stained by rhodamine B and methylene blue) were in contact with each other for 12 h at 25 °C, the healed PHEAA-Car hydrogel could hold a weight of 18 g, suggesting the PHEAA-Car hydrogel possessed a self-healing property.

Then, the effect of the weight ratio of PHEAA to Car on the HE of PHEAA-Car hydrogel was investigated. As displayed in Figure 7b,c, when the weight ratios of PHEAA to Car were in the range of 39:1–37:3, the PHEAA-Car hydrogel possessed a similar self-healing efficiency (13.15–13.48%). However, when the weight ratio of PHEAA to Car was 36:4, the HE of the H_36_C_4_K_6_ hydrogel sharply decreased to 8.89%. This result was probably because the elastic modulus of the H_36_C_4_K_6_ hydrogel was much higher than those of the H_39_C_1_K_6_, H_38_C_2_K_6_, and H_37_C_3_K_6_ hydrogels, leading to a decrease in the motion ability of the molecular chain and a lower HE. The above results prove that the PHEAA-Car hydrogel had a self-healing property at 25 °C.

**Figure 8:**
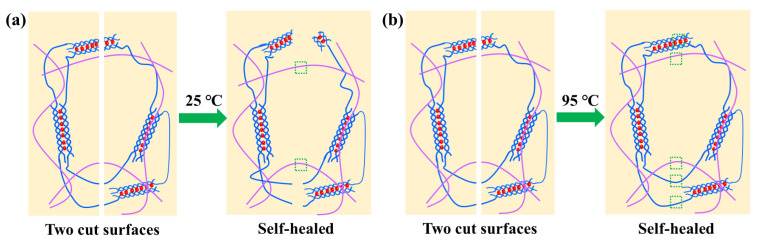
Schematics for the self-healing mechanism of PHEAA-Car hydrogel at (**a**) 25 °C and (**b**) 95 °C.

At a high temperature, the broken Car hydrogel can turn to sol state, and after cooling, it can heal and reform to bulk hydrogel. Therefore, the self-healing performance of the PHEAA-Car hydrogel was also tested at 95 °C. As shown in Figure 7d,e, the tensile stress, elongation at break, and self-healing efficiency increased with an increase of the healing time. The tensile strength, elongation at break, and self-healing efficiency of the H_38_C_2_K_6_ hydrogel at 95 °C for 6 h was 0.47 MPa, 165%, and 10.53%, respectively. As the healing efficiency of the hydrogel at 4 h and 6 h was similar, the self-healing performance of the PHEAA-Car hydrogel with different ratios of PHEAA to Car at 95 °C was investigated at 4 h. As shown in Figure 7f, the elongation at breaks of the healed H_37_C_3_K_6_ and H_36_C_4_K_6_ hydrogels were lower than the H_39_C_1_K_6_ and H_38_C_2_K_6_ hydrogels due to their high elastic modulus. The elongation at breaks of all the healed HEAA-Car hydrogels were greater than 40%. The self-healing property of the hydrogel at 95 °C was only a little better than that at 25 °C, which was probably because under high temperature conditions, the hydrogel lost a considerable amount of water, causing it to be more brittle and easier to break upon stretching. In fact, in the self-healing experiment, we indeed observed that the PHEAA-Car hydrogel appeared in a dehydrated state after healing at 95 °C. The schematics for the self-healing mechanism of the PHEAA-Car hydrogel at 25 °C and 95 °C are shown in Figure 8.

Next, we compared the healing result of the PHEAA-Car hydrogel at 25 °C with other Car-based hydrogels in the literature at high temperatures, and we found that the fracture strain of the healed PHEAA-Car hydrogel at 25 °C is much higher than most of the reported healed Car-based hydrogels at 95 °C (Table 1), which further confirms that the PHEAA-Car hydrogel has advantages in self-healing performances and healing temperatures.

### 3.5. Conductivity of Hydrogel

**Figure 9:**
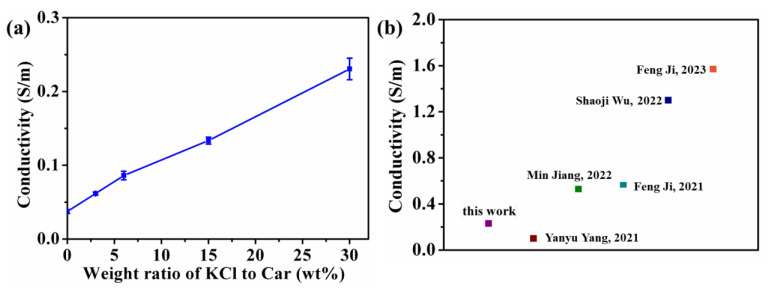
(**a**) The conductivity of PHEAA-Car hydrogel with different weight ratios of KCl to Car. (**b**) Comparison result of conductivity of PHEAA-Car hydrogel with other PHEAA-based hydrogels in the literature [26,29,42,43,44].

KCl can give PHEAA-Car hydrogels conductivity. As displayed in Figure 9a, the conductivity of the PHEAA-Car hydrogel increased from 0.037 S/m to 0.231 S/m by increasing the weight ratio of KCl to Car from 0 wt% to 30 wt%. Moreover, we compared the conductivity of the PHEAA-Car hydrogel with other PHEAA-based hydrogels in the literature. As shown in Figure 9b, the conductivity of the PHEAA-Car hydrogel was at a relatively low level because the concentration of KCl in this work was at a relatively low level. In fact, in the experiment, we found that the Car network could not be formed at a high KCl concentration, so the weight ratio of KCl to Car was determined in the range of 0–30 wt%.

### 3.6. Sensing Performance of Hydrogel

**Figure 10:**
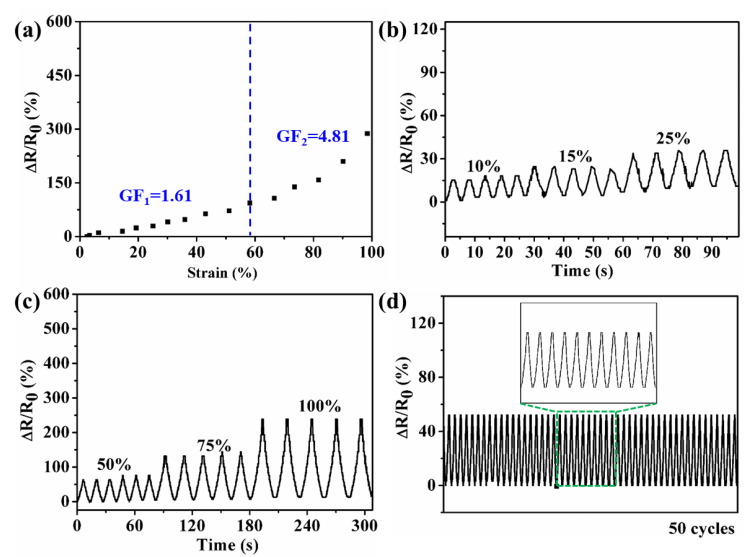
(**a**) Relative resistance variations and corresponding GF values under changed strains (0–100%), (**b**,**c**) relative resistance variations with cyclic loading/unloading of different strains, and relative resistance variations (**d**) upon stretching to 40% strain for 50 cycles of H_37_C_3_K_6_ hydrogel-based strain sensor.

GF, which is defined as the ratio of relative resistance changes to the applied strain, is often used to evaluate the sensitivity of stretchable sensors [45,46,47]. As shown in Figure 10a, when the strain was 100%, the Δ*R*/*R*_0_ was 287%, and the GF values were 1.61 and 4.81 in the strain ranges of 0–58%, 58–100%, respectively. These results suggest that the PHEAA-Car hydrogel-based strain sensor had sensitivity. Meanwhile, the PHEAA-Car hydrogel-based strain sensor was able to monitor and output different electrical signals at different strains (10–25% and 50–100%) with repeatability (Figure 10b,c). Moreover, the PHEAA-Car hydrogel-based strain sensor outputted repeatable signals at a strain of 40% for 50 cycles (Figure 10d), indicating the stability of the H_37_C_3_K_6_ hydrogel.

**Figure 11:**
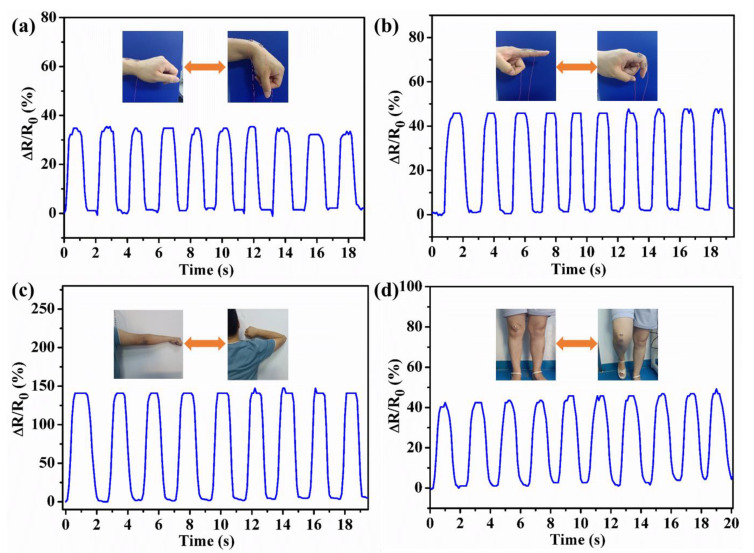
The applications of H_37_C_3_K_6_ hydrogel. Relative resistance variations of detecting joint movements: (**a**) wrist, (**b**) finger bending, (**c**) elbow, and (**d**) knee.

Based on integrated performances such as stretchability, transparency, self-healing, and strain sensitivity, the PHEAA-Car hydrogel was designed as a strain sensor. Figure 11a–d show the joint motions could be precisely detected. Meanwhile, wrist, finger, elbow, and knee bending showed different waveforms as the deformation for each joint was different. The motion of the elbow produced the highest peaks due to the largest deformation. All these results indicate that the PHEAA-Car hydrogel-based strain sensor exhibited desirable applications in wearable devices.

## 4. Conclusions

In summary, we developed a multifunctional PHEAA-Car hydrogel with stretchability (1145%), transparency (92%), and self-healing properties. The driving forces for physical gelation were hydrogen bonds, ion bonding, and electrostatic interactions. Furthermore, the PHEAA-Car hydrogel possessed sensing performances, which could monitor different joint movements. Taking advantage of these features, the PHEAA-Car hydrogel has the prospect of a bright future in the field of flexible strain sensors, electronic skins, and other related fields.

## Figures and Tables

**Table 1:** The fracture strain of the healed carrageenan-based hydrogels.

Composition	Healing Condition	Strain of the Healed Gel	Reference
PHEAA-Car	25 °C	152%	This work
PAAm-Car-KCl	90 °C	83%	[33]
PAAm-Car-KCl	95 °C	52%	[34]
PAAm-Car-EG/Gl-KCl	85 °C	150%/170%	[40]
PAAm-Car-EG/Gl-KCl	90 °C	85%	[41]

## Data Availability

Data sharing is not applicable to this article.
